# Long-acting *Erwinia chrysanthemi*, Pegcrisantaspase, induces alternate amino acid biosynthetic pathways in a preclinical model of pancreatic ductal adenocarcinoma

**DOI:** 10.1186/s40170-024-00346-2

**Published:** 2024-06-30

**Authors:** Dominique Bollino, Kanwal Hameed, Anusha Bhat, Arveen Zarrabi, Andrea Casildo, Xinrong Ma, Kayla M Tighe, Brandon Carter-Cooper, Erin T. Strovel, Rena G. Lapidus, Ashkan Emadi

**Affiliations:** 1 Department of Medical Oncology, West Virginia University School of Medicine, Morgantown, WV USA; 2 West Virginia University Cancer Institute, Morgantown, WV USA; 3 University of Maryland Marlene and Stewart Greenebaum Comprehensive Cancer Center, Baltimore, MD USA; 4 Department of Pathology, University of Maryland School of Medicine, Baltimore, MD USA; 5 Department of Medicine, University of Maryland School of Medicine, Baltimore, MD USA

**Keywords:** Pancreatic cancer, KPC, Asparaginase, Glutamine, Asparagine, Pegcrisantaspase, Crisantaspase

## Abstract

**Background:**

Pancreatic ductal adenocarcinoma (PDAC) is an aggressive disease without meaningful therapeutic options beyond the first salvage therapy. Targeting PDAC metabolism through amino acid restriction has emerged as a promising new strategy, with asparaginases, enzymes that deplete plasma glutamine and asparagine, reaching clinical trials. In this study, we investigated the anti-PDAC activity of the asparaginase formulation Pegcrisantaspase (PegC) alone and in combination with standard-of-care chemotherapeutics.

**Methods:**

Using mouse and human PDAC cell lines, we assessed the impact of PegC on cell proliferation, cell death, and cell cycle progression. We further characterized the in vitro effect of PegC on protein synthesis as well as the generation of reactive oxygen species and levels of glutathione, a major cellular antioxidant. Additional cell line studies examined the effect of the combination of PegC with standard-of-care chemotherapeutics. In vivo, the tolerability and efficacy of PegC, as well as the impact on plasma amino acid levels, was assessed using the C57BL/6-derived KPC syngeneic mouse model.

**Results:**

Here we report that PegC demonstrated potent anti-proliferative activity in a panel of human and murine PDAC cell lines. This decrease in proliferation was accompanied by inhibited protein synthesis and decreased levels of glutathione. In vivo, PegC was tolerable and effectively reduced plasma levels of glutamine and asparagine, leading to a statistically significant inhibition of tumor growth in a syngeneic mouse model of PDAC. There was no observable in vitro or in vivo benefit to combining PegC with standard-of-care chemotherapeutics, including oxaliplatin, irinotecan, 5-fluorouracil, paclitaxel, and gemcitabine. Notably, PegC treatment increased tumor expression of asparagine and serine biosynthetic enzymes.

**Conclusions:**

Taken together, our results demonstrate the potential therapeutic use of PegC in PDAC and highlight the importance of identifying candidates for combination regimens that could improve cytotoxicity and/or reduce the induction of resistance pathways.

**Supplementary Information:**

The online version contains supplementary material available at 10.1186/s40170-024-00346-2.

## Background

Pancreatic ductal adenocarcinoma (PDAC) is an aggressive and lethal malignancy that is predicted to be the second leading cause of cancer-related deaths in the United States by 2030 [1]. The 5-year survival rate for localized, locally advanced, and metastatic cases is approximately 42%, 14% and 3%, respectively [2]. Due to the lack of early detection methods, the majority of patients are diagnosed at an advanced, disseminated stage when curative intent through surgery is no longer attainable. Standard chemotherapy regimens consist of FOLFIRINOX (5-fluorouracil [5FU], leucovorin, irinotecan [liposomal or traditional formulations] and oxaliplatin) or gemcitabine plus nab-paclitaxel [3]; however their anti-tumor effects are remarkably lower than for other solid tumors, and there is currently no chemotherapeutic regimen that provides meaningful clinical outcome beyond the first salvage therapy, underscoring the urgent need for novel therapeutic strategies.

Depletion of amino acids asparagine and glutamine has emerged as a therapeutic approach for cancers, including PDAC, that are dependent on an exogenous (e.g. blood) amino acid supply for survival and proliferation. L-asparaginases are enzymes that primarily catalyze the hydrolysis of asparagine to aspartate and through their simultaneous glutaminase activity, glutamine to glutamate; thereby reducing circulating levels of both asparagine and glutamine. Clinically available asparaginase is derived from 2 bacterial sources: *Escherichia coli* and *Erwinia chrysanthemi* (called crisantaspase), with higher glutaminase activity, hence, more profound glutamine depletion, reported for crisantaspases [4, 5]. The use of asparaginase is well-established as part of multi-agent chemotherapeutic regimens in pediatric and adult acute lymphoblastic leukemia (ALL). Asparaginases are also being investigated in solid tumors with asparagine and glutamine dependencies, including PDAC. A Phase 2 clinical trial demonstrated that treatment with Eryaspase (asparaginase encapsulated inside a donor-derived red blood cell) in combination with chemotherapy for the second-line treatment of patients with metastatic pancreatic cancer reduced the risk of death by 40% compared to treatment with chemotherapy alone [6, 7]. Eryaspase, however, uses an *E. coli* derived asparaginase with lower glutaminase activity, is expensive to produce, and requires donor blood type matching and blood transfusion for administration. Pegcrisantaspase (PegC), a PEGylated (having polyethylene glycol attached) crisantaspase does not require donor matching or blood transfusion and would therefore potentially represent a favorable therapeutic strategy. Given the emerging role of targeting glutamine/asparagine metabolism in pancreatic cancer, we sought to investigate the potential of PegC as an anti-pancreatic cancer agent both in vitro and in vivo and whether it could enhance the anti-cancer activity of standard-of-care chemotherapeutics.

## Methods

### Cell culture and reagents

The human pancreatic adenocarcinoma cell lines Panc10.05, SW-1990, BxPC3, Capan 2, CFPAC-1, HPAF-II, and AsPC-1 were purchased from ATCC (Manassas, VA). MiaPaCa-2 were kindly provided by the Translational Lab Shared Service at University of Maryland Baltimore, and the murine PDAC line KPC were kindly provided by Dr. Javed Mahmood (University of Maryland Baltimore). KPC, SW-1990, and MiaPaCa2 cells were cultured in DMEM supplemented with 10% fetal bovine serum (FBS). BxPC3 and AsPC-1 cells were cultured in RPMI-1640 supplemented with 10% FBS and Panc10.05 were cultured in RPMI-1640 supplemented with 15% FBS. Capan-2 cells were cultured in McCoy’s 5a Medium Modified supplemented with 10% FBS and HPAF-II cells were cultured in EMEM supplemented with 10% FBS.

PegC was provided by Jazz Pharmaceuticals. Gemcitabine, 5-fluorouracil (5-FU), and irinotecan were purchased from Sigma Aldrich (Burlington, MA). Oxaliplatin was purchased from Tocris (Bristol, UK) and paclitaxel was purchased from Enzo Life Sciences (Villeurbanne, France). INK128 and cobimetinib were purchased from Med Chem Express (Monmouth Junction, NJ).

### Cell proliferation assay

Cell lines were seeded into 96-well plates the afternoon prior to treatment. Optimal seeding density was determined for each cell line. For single agent PegC dose response, Peg-C was serially diluted in growth medium and added to cells approximately 18 h after plating. For PegC-chemotherapy combinations, the chemotherapy drug was serially diluted in vehicle then added to either normal growth media or media with a low dose (IC10-30) of PegC. Each condition had at least 4 technical replicates and the assay was repeated at least 3 times. After 72 h, water-soluble tetrazolium (WST-1) (Clontech, Mountain View, CA) was added and plates were read using a BioTek Synergy H1 plate reader (BioTek, Winooski, VT) after 4 additional hours of incubation at 37oC. Data were analyzed and graphed using GraphPad Prism Software (GraphPad, La Jolla, CA) and IC_50_ concentrations were calculated.

### Cell cycle analysis

Cells were seeded into T25 cell culture flasks and treated the next day with either vehicle or increases doses of PegC. After 24 h, cells were harvested, washed in PBS, then fixed/permeabilized in ice cold 70% ethanol for 2 h at 20 °C. Cells were washed twice in cold PBS then resuspended in staining buffer (PBS with 0.5% BSA and 2 mM EDTA) with RNase (100 µg/mL) and propidium iodide. After a 1-h incubation at 4 °C, samples were run on the BD FACS Canto II (BD Biosciences, San Jose, CA) and data was analyzed using FCS Express Version 7 (De Novo Software, Pasadena, CA).

### Western blot analysis

Cells were lysed with radioimmunoprecipitation assay (RIPA) buffer (Millipore, Burlington, MA) supplemented with protease and phosphatase inhibitor cocktails (Sigma Aldrich). Excised tumor tissues were lysed using tissue extraction reagent (ThermoFisher Scientific, Waltham, MA) supplemented with protease and phosphatase inhibitor cocktails. Lysates were incubated on ice for 10 min then centrifuged at 10,000 g at 4 °C for 15 min. Protein content of lysates was determined using Bradford Dye Reagent (BioRad, Hercules, CA), and lysates were separated by 4–15% polyacrylamide gels (BioRad), then transferred onto polyvinylidene difluoride (PVDF) membranes (BioRad). Membranes were blocked with either 5% non-fat milk or bovine serum albumin in tris-buffered saline with 0.1% Tween 20 (TBST), incubated with primary antibodies at 4 °C overnight, then incubated with HRP-conjugated secondary antibody for 1 h. Bands were visualized using Clarity Western Enhanced Chemiluminescence (ECL) substrate (BioRad) or SuperSignal West Pico Plus ECL substrate (Thermo Fisher Scientific). Densitometric analyses were performed using ImageJ (NIH).

Antibodies against actin, GAPDH, ASNS, PHGDH, PSAT, phosphorylated/total 4EBP1 and eIF4E, and horseradish peroxidase (HRP)-conjugated secondary antibodies were purchased from Cell Signaling Technology (Danvers, MA). Antibodies against eIF4A and eIF4B were purchased from Santa Cruz Biotechnology (Dallas, TX).

### Puromycin incorporation assay (SUnSET assay)

SUnSET assay was performed as per manufacturer’s recommendations (Kerafast, Boston, MA). Briefly, cells were treated with vehicle control or PegC at 0.01, 0.1, or 1 IU/mL for 16 h then incubated with puromycin (1 µg/mL) for 20 min. Following incubation, cells were washed with PBS then lysed with RIPA buffer. Protein lysates were separated by SDS-PAGE and probed with anti-puromycin antibody (Kerafast), then striped and re-probed for actin as a loading control.

### Measurement of glutathione, glutamate, and reactive oxygen species

Glutathione (GSH) was measured using the GSH-Glo Glutathione Assay kit (Promega), a luminescence-based assay for detecting and quantifying GSH, based on the conversion of a luciferin derivative into luciferin in the presence of GSH. Briefly, cells were seeded into 96-well plates and treated after approximately 18 h with either vehicle or increasing doses of PegC (ranging from 0.01 to 1 IU/mL for SW1990, Panc10.05, and MiaPaCa2 cells and from 1 to 10 IU/mL for KPC cells) After 24 h, culture media was removed, and the cells were incubated with the GSH-Glo reagent. After a 30-minute (min) incubation, reconstituted luciferin detection reagent was added to the plate. Luminescence was measured after 15 min using a Biotek Synergy H1 plate reader. Intracellular glutamate was measured using the Glutamine/Glutamate Glo Assay kit (Promega), a luminescence-based assay for rapid detection of glutamine/glutamate in biological samples. Briefly, KPC and SW1990 were seeded into 96-well plates then treated the next day with vehicle or increasing concentrations of PegC. After 24 h, culture media was removed, and glutamate was detected as per manufacturer’s instructions.

For detection of reactive oxygen species (ROS), cells plated and treated as above were preloaded with 2′,7′-dichlorodihydrofluorescein diacetate (H2DCFA, Life Technologies) at a final concentration of 2 µM and incubated at 37 °C in the dark for 25 min. Cells were washed with PBS and treated with either vehicle, PegC (1, 5, or 10 IU/mL), or hydrogen peroxide as a positive control in phenol red-free DMEM or RPMI plus 10% FBS. Cells were incubated in the dark at 37 °C and measurement of ROS was performed with a Bio-Tek Synergy H1 plate reader at the 0, 1, 2, 4, and 6 h time points.

### In vivo mouse studies

For all studies, mice were housed under pathogen-free conditions in a University of Maryland Baltimore Association for Assessment and Accreditation of Laboratory Animal Care (AAALAC)-accredited facility. All experiments were conducted in compliance with Public Health Service (PHS) guidelines for animal research and approved by the University of Maryland Baltimore Institutional Animal Care and Use Committee. For the in vivo study examining the effect of irinotecan and PegC alone and in combination on tumor growth, KPC cells (3 × 10^6^) were injected subcutaneously into the flank of female C57BL/6 mice. Tumor volume was measured by calipers and when tumors were palpable (~ 160 mm^3^; 11 days post injection), mice were sorted into groups and treatment was initiated. Mice were treated with either vehicle (*n* = 8), irinotecan (*n* = 8; 75 mg/kg, IV, 1X/week), PegC (*n* = 7; 300 IU/kg, IV, 1X/week) or irinotecan in combination with PegC (*n* = 8). Mice were euthanized when endpoint criteria (including tumor ulceration/necrosis and body weight loss ≥ 18%) were met. The study was terminated on day 21 since the majority of the mice in 2 of the 4 treatment groups (vehicle and irinotecan) reached endpoint criteria. Tumors were excised upon euthanasia and frozen at -80 °C. Plasma was isolated from whole blood collected in K2EDTA tubes (Greiner Bio-One, Monroe, NC).

To determine the effect of PegC treatment on tumor growth, KPC cells (3 × 10^6^) were injected subcutaneously into the flank of female C57BL/6 mice. When tumors were palpable (150mm^3^; 9 days post injection), mice were treated with either vehicle (*n* = 7) or PegC at a dose of 500 (*n* = 6) or 700 IU/kg (*n* = 7) intravenously once weekly. Mice were weighed 5 times a week and tumor volumes were measured by caliper over time. Mice were euthanized when endpoint criteria were met and the study was terminated on day 30 when all vehicle-treated mice reached endpoint. Tumors were excised upon euthanasia and frozen at -80 °C. Plasma was isolated from whole blood collected in K2EDTA tubes.

### Plasma amino acid measurement

Plasma was isolated from whole blood and delivered on wet ice to the University of Maryland Pathology Associates Biochemical Genetics Laboratory for plasma quantitative amino acid (AA) analysis. Within 40 min of collection, the plasma was separated by centrifugation (1100 g × 5 min at 4 °C) and frozen at -20 °C until analysis. Free AA concentrations were measured using a Biochrom 30 or Biochrom 30 + Amino Acid Analyzer (Biochrom Ltd., Cambridge, UK) by cation-exchange chromatography and ninhydrin detection according to the manufacturer’s instructions. Results were quantified using commercially available calibration standards and normalized to the internal standard, s-2-aminoethylcysteine and reported as µmol/L (µM). Standard curves were generated for each AA and AAs were quantified. Two quality control standards were evaluated as an unknown at the beginning of each set of sample runs.

### Statistics

For in vitro studies, data are expressed as means ± standard error of the mean (SEM) and independent t-tests were used to determine significance. For both in vitro and in vivo experiments, statistical analyses to compare vehicle and PegC treated groups were performed using unpaired t- tests and the level of significance were presented as ****p < 0.0001, ***p < 0.001, ** *p* < 0.01, *p < 0.05, ns = not significant. Analysis of variance (ANOVA) was used to compare differences among multiple groups and was followed by Bonferroni’s post hoc correction. Statistical analyses were performed using GraphPad Prism Version 8.1.2 (La Jolla, CA).

## Results

### PegC has potent single-agent anti-proliferative activity against pancreatic adenocarcinoma cell lines

**Fig. 1:**
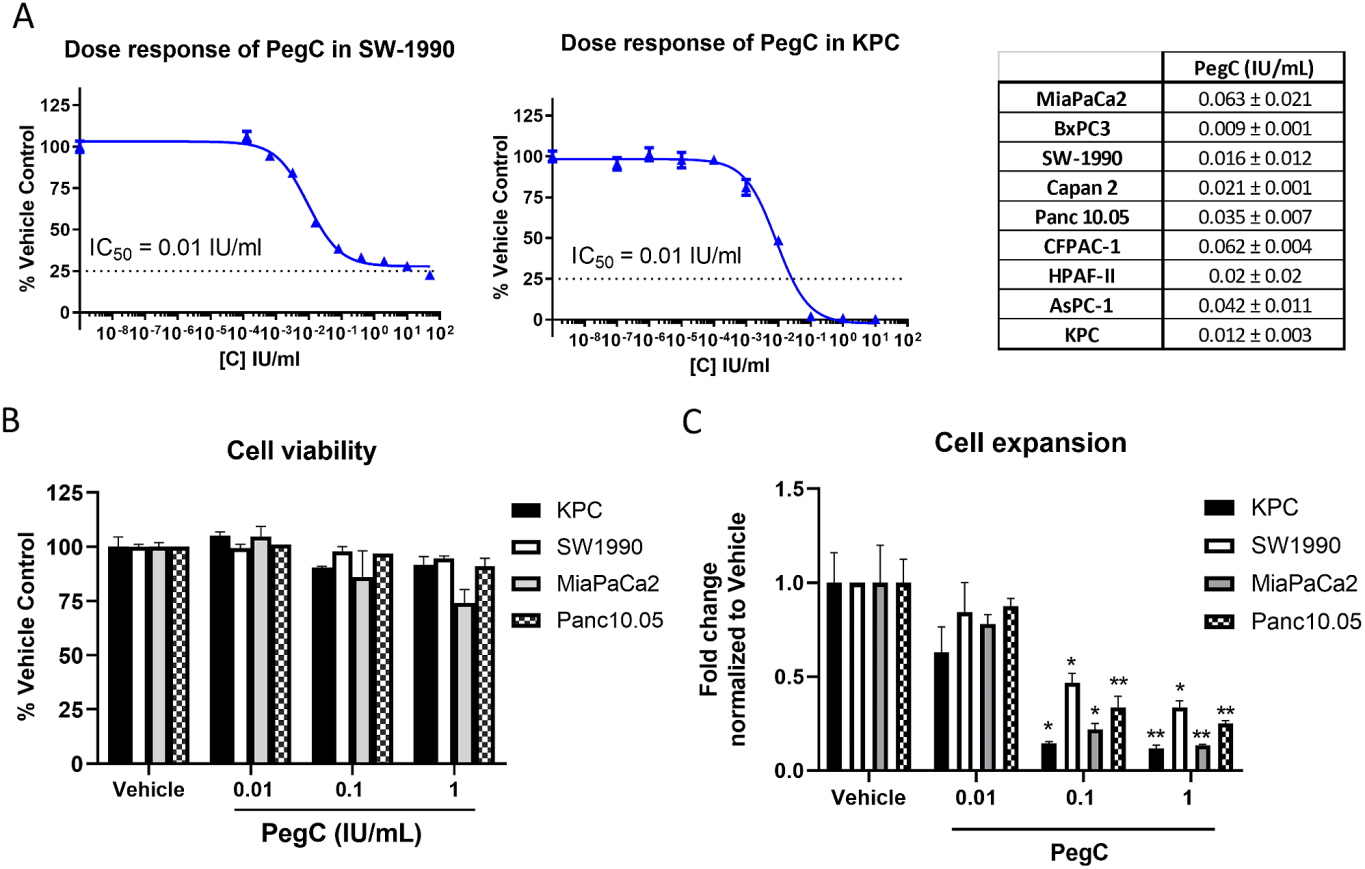
PegC inhibits PDAC cell growth **(A)** KPC and SW-1990 cells were treated with serially-diluted PegC and cell proliferation was measured 72 h post-treatment using WST-1. Dose-response curves were generated and IC_50_ values were calculated using GraphPad Prism. The table shows PegC IC_50_ values ± standard deviation (SD) for all 9 PDAC cell lines tested. **(B)** The percentage of viable cells after PegC treatment for 72 h was determined using trypan blue exclusion. Data are expressed as the percentage of the vehicle control. **(C)** After 72 h PegC treatment, the cell culture expansion was calculated by dividing the total number of viable cells by the number of cells initially plated. Data are expressed as fold change normalized to vehicle control (fold change for each treatment group was divided by the fold change for the vehicle-treated cells). Statistical analyses to compare vehicle and PegC treated groups were performed using unpaired t- tests. **p < 0.01, *p < 0.05

The anti-proliferative effect of PegC was tested in eight human PDAC cell lines (MiaPaCa2, SW-1990, BxPC3, AsPC-1, HPAF-II, Capan 2, CFPAC-1, and Panc10.05) and one murine line (KPC). Treatment with single-agent PegC decreased in vitro proliferation of PDAC cell lines in a concentration-dependent manner, with IC_50_s ranging from 0.009 to 0.063 international units per milliliter (IU/mL) (Fig. 1A). Of note, these IC_50_s are clinically meaningful as a nadir plasma asparaginase activity of 0.1 IU/mL has been accepted as a clinically effective and pharmacologically achievable threshold in many treatment protocols, clinical research and by regulatory agencies [8–13].

We hypothesized that the higher glutaminase activity of PegC compared to *E. coli*-derived asparaginases may be advantageous as it was recently reported that the anti-tumor activity of asparaginases in PDAC is dependent on its glutaminase activity [14]. To establish the contribution of glutamine depletion to PegC-mediated anti-PDAC activity, we treated PDAC cell lines with PegC in either normal growth media with 2 mM glutamine or media without glutamine supplementation. Without glutamine in the media, the anti-proliferative activity of PegC was potentiated, as indicated by a decrease in the IC_50_s, confirming that the glutaminase activity of PegC is important for mediating its effect against PDAC cells (Supplemental Fig. S1).

To determine if PegC only halts the proliferation of PDAC cells or also induces PDAC cell death, trypan blue exclusion was performed on PDAC cell cultures treated with increasing doses of PegC for 72 h (h). Compared to a vehicle control, PegC did not significantly increase the proportion of dead cells in any of the cell lines tested (Fig. 1B). This result was confirmed by staining PegC-treated cells with propidium iodide (Supplemental Fig. S2A). PegC did, however, impact PDAC cell culture growth as indicated by the reduction in fold expansion of PegC-treated PDAC cell cultures (Fig. 1C), suggesting that PegC inhibits cell growth and expansion but does not induce cell death.

Furthermore, we sought to evaluate the impact of PegC on cell cycle progression in KPC and SW1990 cells by labeling total DNA content with propidium iodide and performing flow cytometry. PegC treatment stalled KPC cells in the G1 phase of the cell cycle and in SW1990 cells, there was a significant decrease of cells in the S phase and a concordant increase in cells in the G2/M phase, suggesting G2/M phase arrest (Supplementary Fig. S3B).

### PegC inhibits global protein synthesis in PDAC cells

**Fig. 2:**
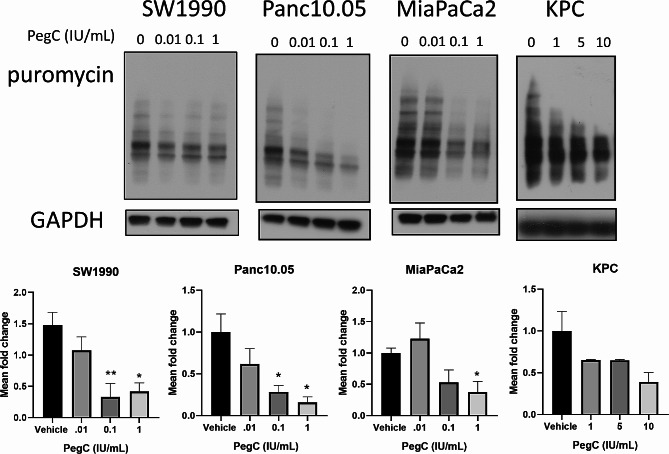
PegC inhibits protein synthesis. PDAC cells were treated with PegC at the indicated doses for 16 h (KPC for 48 h) followed by 20 min of incubation with puromycin (1 µg/mL) and then lysed. Cell lysates were subjected to immunoblotting with the anti-puromycin antibody (SUnSET [surface sensing of translation] assay). GAPDH was used as a loading control. The bar diagram represents densitometric quantification of three in-dependent experiments normalized to the vehicle control. *p < 0.05

Eukaryotic cells perform both cap-dependent and cap-independent (internal ribosome entry site [IRES]-mediated) mRNA translation [15]. A key factor in the initiation of cap-dependent translation is the availability of eIF4E to participate in the eIF4F initiation complex through binding 5´ mRNA caps [16], whereas IRES/cap-independent translation relies on eIF4A and eIF4B and is independent of eIF4E [17, 18]. eIF4E activity can be regulated by both MAPK (Mitogen-activated protein kinases) and mTOR (mammalian target of rapamycin) signaling pathways [19]. mTOR activation leads to the phosphorylation/deactivation of the eIF4E repressor protein, 4EBP1. When mTOR signaling is inhibited, unphosphorylated 4EBP1 binds eIF4E and prevent cap-binding complex formation [19]. We previously reported that PegC, in combination with the Bcl2 inhibitor venetoclax, inhibits protein synthesis through interference with cap-dependent mRNA translation downstream of mTOR signaling in acute myeloid leukemia (AML) [17, 20]. To determine if the observed PegC-mediated growth inhibition is related to inhibition of mTOR signaling and protein synthesis in PDAC, we first assessed global protein synthesis through puromycin incorporation. KPC, SW-1990, MiaPaCa2, and Panc10.05 cells were treated with increasing concentrations of PegC followed by a short exposure to puromycin. Puromycin incorporation into nascent peptides was detected by probing whole cell lysates with anti-puromycin antibody. In all four cell lines tested, PegC inhibited protein synthesis in a dose-dependent manner (Fig. 2). Of note, inhibition of protein synthesis in the murine KPC cell line required higher PegC concentrations than in the human PDAC cell lines, which may be due to interspecies metabolic differences.

To determine if this reduction in protein synthesis can be attributed to an inhibition of cap-dependent mRNA translation downstream of mTOR signaling, we examined the phosphorylation status of mTOR substrate 4EBP1 and its binding target, eIF4E. In contrast to our published findings in AML, PegC treatment of PDAC cell lines did not significantly alter 4EBP1 or eIF4E phosphorylation, suggesting that the observed inhibition of protein synthesis was independent of mTOR-regulated cap-dependent translation mechanisms (Supplemental Fig. S3). We next assessed the expression of eIF4A and eIF4B to determine if PegC impacted cap-independent translation and found that PegC treatment did not significantly alter expression of either protein in KPC and SW1990 cells (Supplemental Fig. S4). These findings strongly suggest alternative unidentified mechanism(s) for PegC (and perhaps other asparaginases)-induced reduction in protein synthesis in PDAC.

### PegC reduces levels of intracellular glutamate and the antioxidant glutathione

**Fig. 3:**
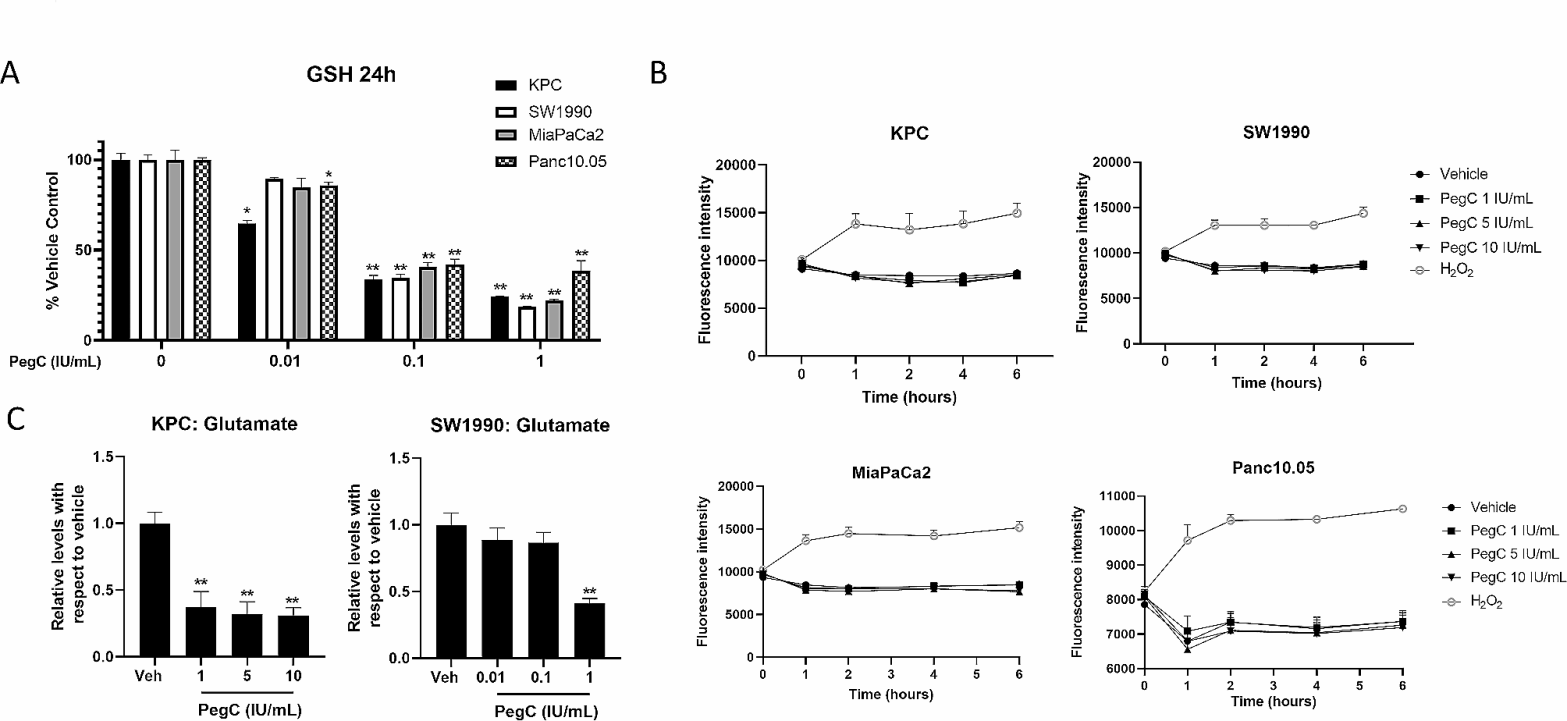
PegC inhibits GSH but does not induce generation of ROS **(A)** PDAC cells were treated with PegC at the indicated doses and 24 h later GSH was measured using a luminescence-based plate assay. Results were normalized to control treated cells and expressed as mean ± SEM (*n* = 3). **(B)** PDAC cells were pre-loaded with ROS sensitive dye H2DCFA then exposed to PegC at the indicated doses or 200 µM H_2_O_2_ as a positive control. ROS levels were measured over time using a plate reader. Results were normalized to control treated cells and expressed as mean ± SEM (*n* = 3). **(C)** KPC and SW1990 cells were treated with the indicated doses of PegC and 24 h later intracellular glutamate was measured using a luminescence-based plate assay. Statistical analyses to compare vehicle and PegC treated groups were performed using unpaired t- tests. **p < 0.01, *p < 0.05. SEM = Standard Error of Mean

The antioxidant glutathione (GSH) is a tri-peptide of cysteine, glycine, and glutamate that is critical for maintaining cellular redox homeostasis [21]. Glutathione exists in reduced (GSH) and oxidized (GSSG) states. Through its oxidation to GSSG, GSH can neutralize reactive oxygen species (ROS). As glutamine is a precursor for glutamate, we aimed to determine if PegC-induced glutamine depletion would impact cellular GSH levels. KPC, SW-1990, MiaPaCa2, and Panc10.05 cells were treated with either vehicle or increasing doses of PegC for 24 h followed by GSH measurement. As shown in Fig. 3A, PegC treatment resulted in a dose-dependent reduction of GSH levels in all cell lines tested at doses of 0.1 and 1 IU/mL.

To determine whether the observed reduction in GSH following PegC exposure was due to an increase in ROS, we measured ROS using H2DCFA, a cell permeable dye that is used as an indicator of ROS. Cells were exposed to increasing doses of PegC alongside hydrogen peroxide (H_2_O_2_) as a positive control, and fluorescence was measured over the course of 6 h. PegC treatment did not increase ROS levels (Fig. 3B), suggesting that the decrease in GSH was the result of decreased GSH production due to glutamine depletion, rather than through oxidation of GSH to GSSG by ROS. To confirm this, we measured intracellular glutamate levels in KPC and SW1990 cells 24 h after PegC treatment and found that, indeed, PegC significantly decreased intracellular glutamate levels (Fig. 3C).

### PegC does not potentiate the effect of standard-of-care chemotherapeutics

**Fig.4:**
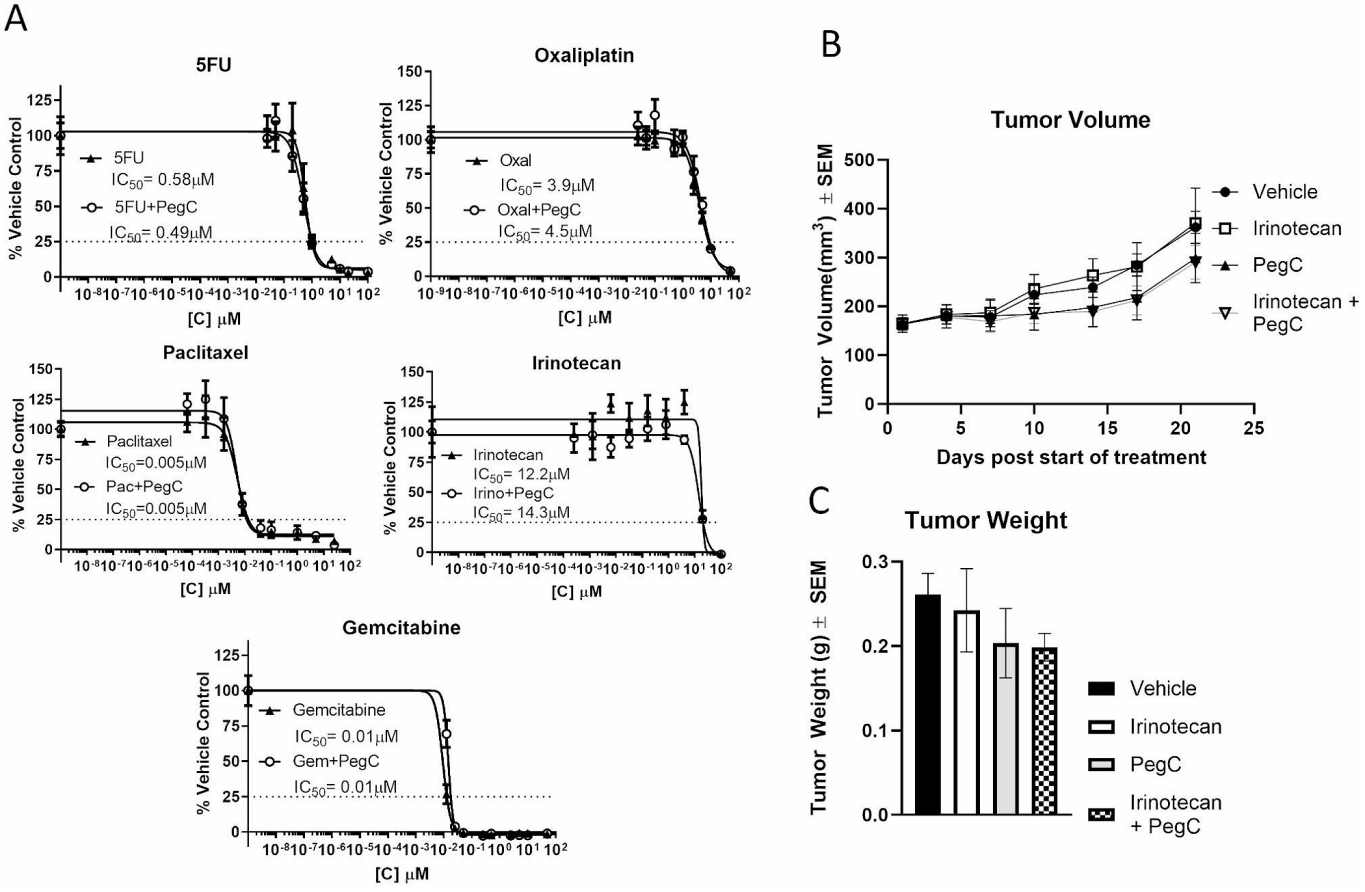
PegC does not potentiate standard-of-care chemotherapies. **(A**) KPC cells were treated with dose curves of the indicated chemotherapy alone or in combination with a low dose (IC_10 − 30_) of PegC. Cell proliferation was measured 72 h after treatment by WST-1. **(B-C)** KPC cells (3 × 10^6^) were injected subcutaneously into the flank of C57BL/6 mice. When tumors reached 150 mm^3^, mice were treated with vehicle, irinotecan (75 mg/kg, IV, 1x/week), PegC (300IU/kg, IV, 1x/week), or irinotecan + PegC. **(B)** Tumor volume over time. **(C)** Tumor weight at study termination

Next, we aimed to determine whether PegC-mediated inhibition of protein synthesis could potentiate the effect of chemotherapeutics commonly used in clinical practice against PDAC. KPC cell cultures were treated with serially diluted gemcitabine, 5FU, oxaliplatin, irinotecan, or paclitaxel alone and in combination with PegC at IC_10_-IC_30_. Addition of PegC did not potentiate the cytotoxic effect of chemotherapy agents (Fig. 4A, and summarized in Supplemental Fig. S5A), suggesting, at least in this model, that the anti-neoplastic effect of PegC against PDAC does not overlap with antimetabolites, alkylators, and topoisomerase or mitotic inhibitors.

Despite the in vitro results, we aimed to test PegC monotherapy and the combination of PegC with a clinically used chemotherapeutic in vivo to evaluate its potential anti-PDAC activity in a pharmacologically relevant model. We used an immunocompetent mouse model (C57BL/6 mice with C57BL/6-derived KPC cell line), since glutamine deprivation/blockade has been reported to enhance anti-tumor T cell responses [22–25]. KPC-tumor bearing mice were treated with either vehicle, PegC (300 IU/kg), irinotecan (75 mg/kg), or PegC plus irinotecan combination and tumor volume was monitored overtime. Compared to the vehicle-treated group, neither irinotecan nor PegC as single agents significantly inhibited tumor volume over time or tumor weight at study termination (Fig. 4B and C). Of note, PegC-treated tumors at the conclusion of the study had a lower mean volume (298 ± 50 mm^3^) compared to vehicle-treated tumors (361 ± 33 mm^3^), indicating that PegC alone can modestly inhibit tumor growth.

Next, we investigated the potential effect of PegC combined with targeted therapies. It has been previously reported that translational reprogramming mediated through MAPK signaling results in adaptation to asparagine restriction [26], suggesting that MAPK pathway inhibition may synergize with PegC. Furthermore, it has been reported that agents targeting the mTOR pathway can lead to significant inhibition of proliferation, differentiation, and tumor progression in PDAC [27]. Thus, we hypothesized that combining PegC with mTOR inhibition would enhance the anti-cancer effect in PDAC models. To test this, we examined the ability of PegC to potentiate the effect of the MEK inhibitor, cobimetinib, and the mTOR inhibitor, INK-128, in KPC and SW1990 cells. We found that addition of PegC did not improve the anti-proliferative effect mediated by mTOR or MEK inhibition in SW1990 and KPC cells (Supplemental Fig. S5B).

### PegC depletes plasma asparagine and glutamine and inhibits PDAC tumor growth in vivo

**Fig. 5:**
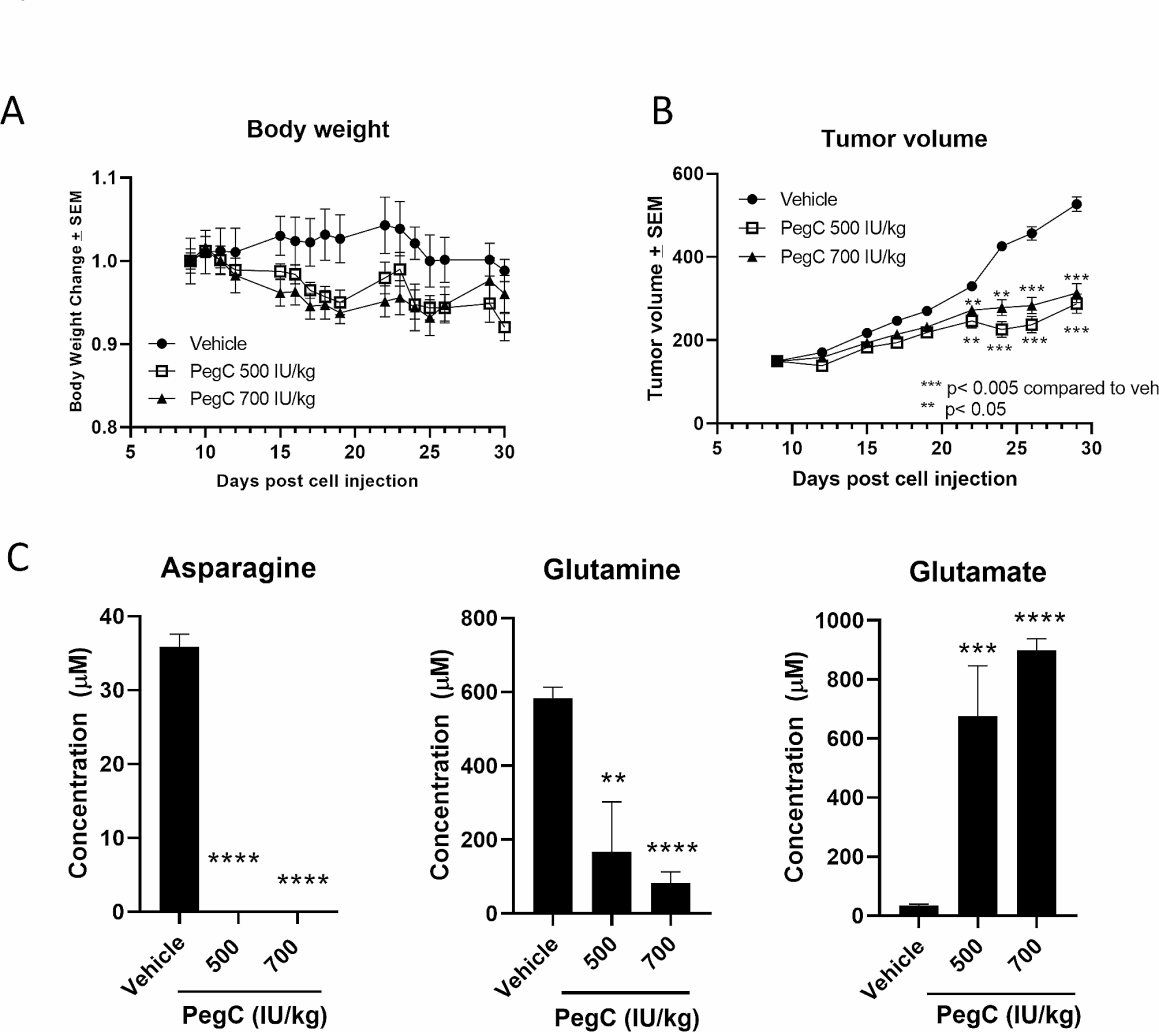
PegC inhibits PDAC tumor growth. KPC cells (3 × 10^6^) were injected subcutaneously into the flank of C57BL/6 mice. When tumors reached 150 mm^3^, mice were treated with vehicle or PegC (500 or 700IU/kg) intravenously once weekly for 3 weeks. **(A)** Percent weight changes versus time. **(B)** Tumor volume over time **(C)** Plasma was isolated from whole blood of euthanized mice and concentrations of glutamine, asparagine, and glutamate levels (µM) were measured. Statistical analyses to compare vehicle and PegC treated groups were performed using unpaired t- tests. ****p < 0.0001, ***p < 0.001, ** *p* < 0.01, *p < 0.05, ns = not significant

To evaluate the dose-response effect of PegC against PDAC cells in vivo, KPC cells were injected subcutaneously, and once tumors were palpable (150 mm^3^), mice were treated with either vehicle or PegC (500 or 700 IU/kg) once weekly. With respect to tolerability, mice in the PegC-treated cohorts did not lose > 10% body weight compared to the vehicle group at any point during the study, suggesting that PegC was tolerable at these increased doses (Fig. 5A). Tumor volume was monitored using calipers overtime. At both 500 and 700 IU/kg, PegC significantly inhibited tumor growth compared to vehicle control starting at day 19 post-treatment until study termination (Fig. 5B).

To evaluate the impact of PegC treatment on plasma amino acid levels, plasma was isolated from euthanized mice as they reached endpoint criteria and amino acid concentrations were measured using HPLC. PegC significantly depleted plasma asparagine from 35.8 ± 1.7 µM in the vehicle-treated mice to undetectable levels in both the 500 IU/kg and 700 IU/kg group. Plasma glutamine was also significantly decreased from 583 ± 30 µM in the vehicle-treated mice to 167 ± 134 µM (*p* = 0.004) and 150 ± 71 µM (*p* < 0.0001) in the 500 and 700 IU/kg groups, respectively. The depletion of glutamine by PegC was accompanied with the expected increase in plasma glutamate (Fig. 5C).

### PegC treatment in vivo induces expression of amino acid response pathway proteins in PDAC tumors

**Fig. 6:**
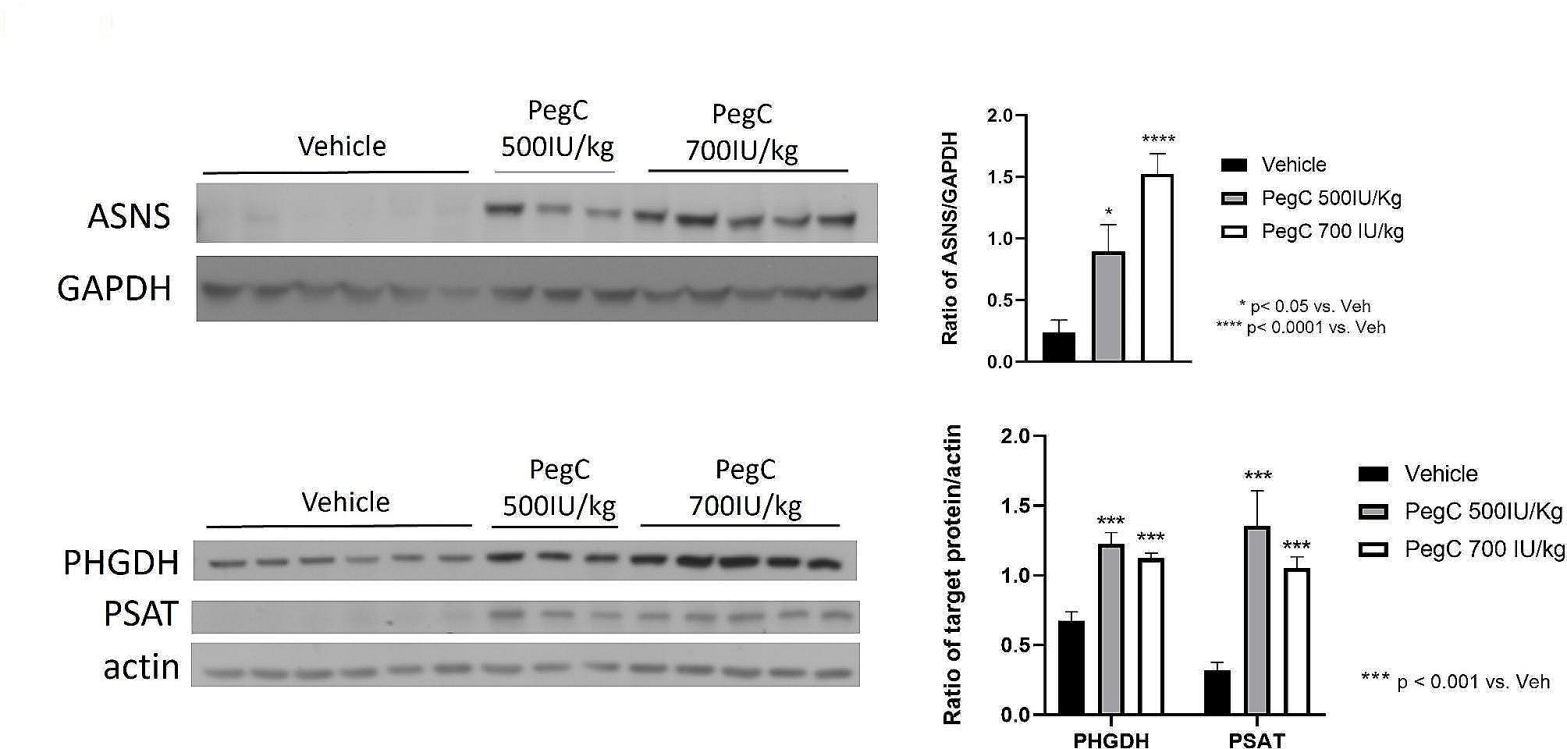
PegC increases tumor expression of serine and asparagine biosynthesis enzymes. Western blot analysis of KPC tumor lysates from mice treated with vehicle or PegC (500 or 700 IU/kg) intravenously once a week. Tumor lysates were probed with the indicated antibodies and actin or GAPDH was used as a loading control. Results were normalized to corresponding vehicle-treated mice and expressed as mean ± SEM. Statistical analyses to compare vehicle and PegC treated groups were performed using unpaired t- tests. ****p < 0.0001, ***p < 0.001, **p < 0.01, *p < 0.05, ns = not significant

Next, we aimed to investigate the impact of PegC treatment on induction of upregulation of pathway(s) that may sustain in vivo tumor resistance. Amino acid restriction triggers the amino acid response (AAR) pathway which initiates with activation of general control nonderepressible 2 (GCN2) kinase. Phosphorylation of the eukaryotic initiation factor 2a (eIF2a) by GCN2 shifts translation from cap-dependent to cap-independent which leads to the translation of transcription factors that promote expression of genes involved in biosynthesis and membrane transport of amino acids to restore homeostasis [28]. Of note, AAR signaling leads to the expression of ASNS as well as the serine biosynthetic enzymes, phosphoglycerate dehydrogenase (PHGDH) and phosphoserine acetyltransferase (PSAT). In ALL, numerous studies have reported that ASNS expression is elevated in asparaginase-resistant leukemia cells compared to asparaginase-sensitive cells [23, 24], and ASNS expression levels have been correlated with asparaginase sensitivity in PDAC cell lines [29]. Vehicle and PegC-treated KPC tumor lysates were immunoblotted for ASNS, PHGDH, and PSAT. PegC treatment significantly induced the expression of both asparagine and serine biosynthesis enzymes in PDAC tumors (Fig. 6).

## Discussion

Here we report that PDAC is sensitive to amino acid depletion mediated by the long-acting crisantaspase, PegC, as evidenced by decreased PDAC cell protein synthesis and proliferation in vitro and the inhibition of PDAC tumor growth in vivo. Importantly, we also found the PegC-mediated induction of alternate amino acid biosynthesis pathways, which may interfere with the therapeutic activity of PegC.

Metabolic reprogramming contributes to tumor development [30] but can, in some cases, introduce specific metabolic liabilities that can be exploited to treat cancer. K-Ras mutation is a signature genetic alteration in pancreatic cancer that drives glutamine dependence through metabolic reprogramming [31], resulting in profound sensitivity to glutamine deprivation [32]. Furthermore, while ASNS is expressed in normal exocrine pancreatic cells, low/null expression of ASNS has been reported in over 70% of resected PDAC tumors [6], suggesting that PDAC cells may be sensitive to asparaginase-mediated glutamine and asparagine depletion [29]. Indeed, all nine PDAC cell lines tested were sensitive to PegC treatment, with IC_50_ values ranging from 0.009 to 0.063 IU/mL, which notably is below the nadir plasma asparaginase activity of 0.1 IU/mL recognized for asparaginase therapeutic activity [8]. By reducing the glutamine concentration in the media, the IC_50_ values for PegC were reduced, demonstrating that the glutaminase activity of PegC is an important mediator of its anti-proliferative effect in PDAC, which is consistent with other reports [14].

We found that while PegC effectively inhibits cell proliferation and culture expansion, it does not significantly induce cell death. We therefore investigated the combination of PegC with conventional cytotoxic chemotherapy (e.g. 5FU, oxaliplatin, gemcitabine, irinotecan, paclitaxel) but did not observe any synergistic or additive benefits to these combinations in vitro or in vivo. Since the anti-cancer activity of these agents depends on active cancer cell DNA replication or cell division, it is plausible that the inhibition of proliferation caused by PegC treatment interfered with cytotoxicity induction. Consistent with our findings, the Phase 3 clinical trial of Eryaspase in combination with gemcitabine + nab paclitaxel or FOLFIRI (folinic acid, fluorouracil, irinotecan) in advanced pancreatic cancer revealed that although patients treated with Eryaspase demonstrated superior disease control compared to patients treated with chemotherapy alone, the difference was not statistically significant, and the trial failed to reach its primary endpoint of overall survival (NCT03665441). In addition to cytotoxic agents, we also investigated the combination of PegC with the MEK inhibitor, cobimetinib, since a synthetic lethal interaction has been reported for MAPK pathway inhibition and asparaginase-mediated asparagine depletion [26]. We did not observe an additive anti-cancer benefit by combining PegC with cobimetinib, however our investigation was limited to in vitro studies.

Our in vivo studies demonstrated that as a single agent, PegC treatment significantly slowed the growth of PDAC tumors in a syngeneic flank model. This is in contrast to a recent report that single-agent *E.coli*-derived asparaginase was unable to inhibit PDAC tumor growth, which confirms the value of utilizing a crisantaspase, which targets both asparagine and glutamine [33]. PegC depleted plasma asparagine to undetectable levels and significantly reduced plasma glutamine. Although PegC exposure decreased the global protein synthesis in PDAC cells; surprisingly, this effect was neither mediated by cap-dependent nor cap-independent mRNA translation. This may be related to eIF4E phosphorylation by MNK downstream of K-Ras in PDAC [34], however the exact mechanism by which PegC inhibits protein synthesis is currently unclear and under investigation.

While amino acid restriction strategies are a promising therapeutic approach in PDAC, metabolic adaptations of cancer cells can limit its effectiveness. Accumulation of uncharged tRNA following amino acid depletion triggers initiation of the AAR pathway to restore amino acid homeostasis. We found that PegC upregulates the amino acid biosynthetic enzymes ASNS, PHGDH, and PSAT in PDAC tumors. We recently published that in addition to the expected decreases in plasma asparagine and glutamine, crisantaspase treatment resulted in a significant increase in plasma levels of serine in AML and PDAC mouse models [29], which cancer cells may be able to take advantage of to sustain growth. Several groups have reported that asparaginase-resistant cells undergo adaptive changes, including increased ASNS expression [35–37], and in ALL, ASNS overexpression is sufficient to confer asparaginase resistance [38]. Furthermore, it has been reported that outside of serine biosynthesis, PHGDH enhances mRNA translation by interacting with eIF4A1 and eIF4E, thereby promoting PDAC tumor growth [39, 40]. The induction of PHDGH and ASNS therefore provides an opportunity for testing the combination of PegC with other targeted agents against PDAC.

Although our use of a syngeneic model provides the advantage of testing therapeutic agents in the context of an intact immune system, a limitation of our study is the use of a flank model rather than an orthotopic model. An orthotopic injection model or genetically engineered mouse model of PDAC would have provided a more clinically accurate platform for testing therapeutic interventions, and efforts are currently underway to develop these models for future studies.

Overall, our findings indicate that while PegC can effectively slow PDAC tumor growth, its anti-cancer activity may be limited by the induction of the AAR pathway and the resulting induction of amino acid biosynthetic enzyme expression. These results lay the groundwork for future studies investigating the combination of PegC with agents that target AAR pathways as a promising therapeutic approach.

### Electronic supplementary material

Below is the link to the electronic supplementary material.

Supplementary Material 1

## Data Availability

No datasets were generated or analysed during the current study.

## Abbreviations

AML: Acute myeloid leukemia
ALL: Acute lymphoblastic leukemia
ASNS: Asparagine synthetase
ECL: Enhanced chemiluminescence
FBS: Fetal bovine serum
GSH: Glutathione
HRP: Horseradish peroxidase
PDAC: Pancreatic ductal adenocarcinoma
PegC: Pegcrisantaspase
PHGDH: Phosphoglycerate dehydrogenase
PVDF: Polyvinylidene difluoride
PSAT: Phosphoserine aminotransferase
RIPA: Radioimmunoprecipitation assay
ROS: Reactive oxygen species
5-FU: 5-fluorouracil
