# Supplementary material for: Long-acting Erwinia chrysanthemi, Pegcrisantaspase, induces alternate amino acid biosynthetic pathways in a preclinical model of pancreatic ductal adenocarcinoma
Source: Cancer Metab. 2024 Jun 30;12:19. doi: 10.1186/s40170-024-00346-2 (PMC11218198; doi:10.1186/s40170-024-00346-2)
Supplement: Supplementary file 1 — Supplementary Material 1 [file 40170_2024_346_MOESM1_ESM.docx]

**Supplementary Materials**

**SUPPLEMENTARY FIGURE 1**

**
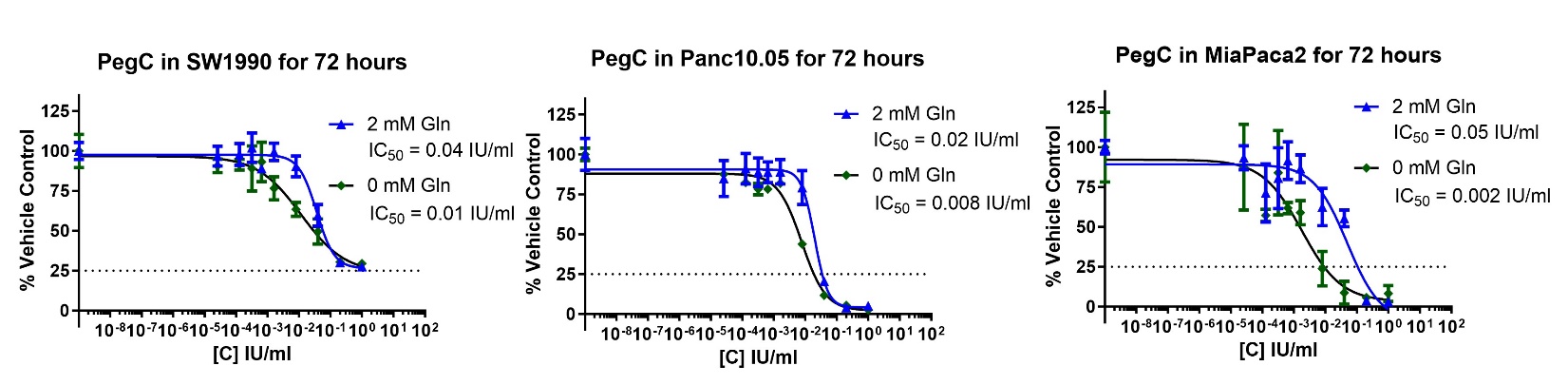
**

**Supplementary Figure 1: Glutamine concentration impacts response to PegC.** PDAC cells were treated with serially-diluted PegC in either normal growth media (2 mM glutamine) or media without glutamine supplementation (0 mM) and cell proliferation was measured 72h post-treatment using WST-1. Dose-response curves were generated and IC_50_ values were calculated using GraphPad Prism.

**
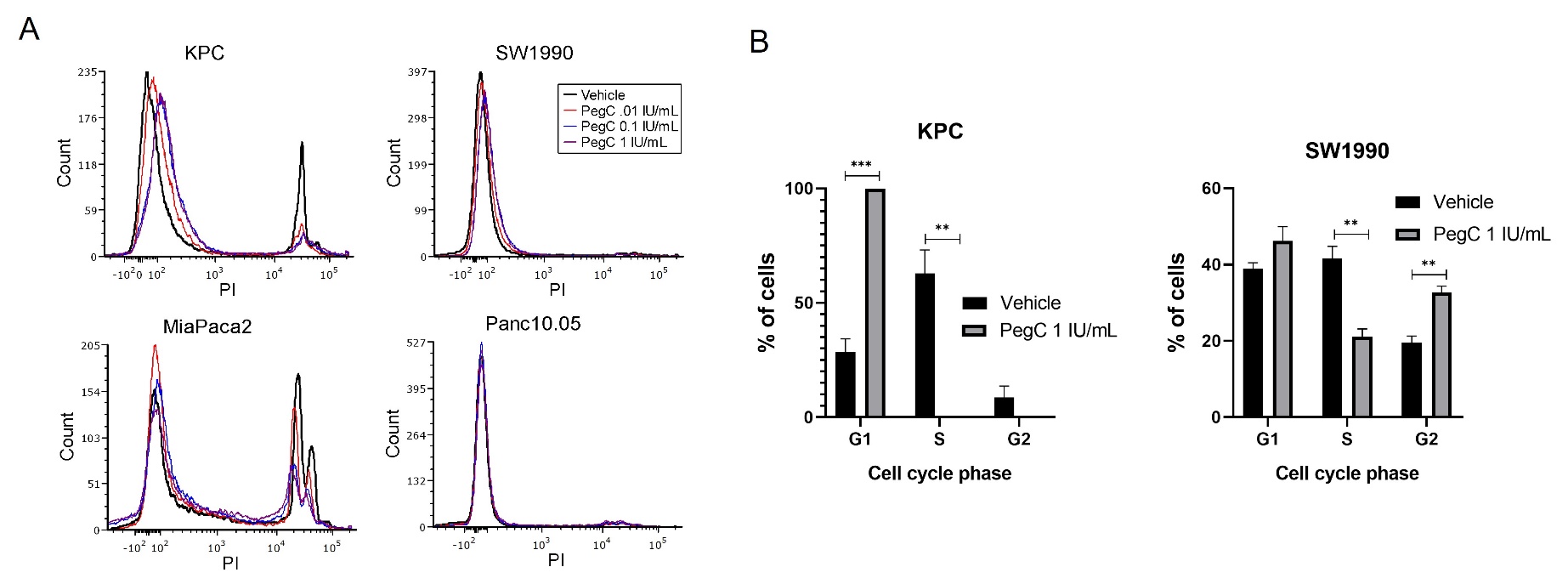
SUPPLEMENTARY FIGURE 2**

**Supplementary Figure 2: PegC does not induce PDAC cell death and impact cell cycle. (A)** PDAC cell lines were treated with vehicle control or the indicated doses of PegC for 72h. After 72 h, harvested cells were stained with propidium iodide and analyzed by flow cytometry to detect dead cells. **(B)** KPC and SW1990 cells were treated with either vehicle or 1 IU/mL PegC for 24h. After 24h, harvested cells were permeabilized and fixed and stained with propidium iodide to measure total DNA content. Cells were analyzed by flow cytometry and the percentage of cells in each cell cycle phase was calculated using FCD Express Version 7.

**SUPPLEMENTARY FIGURE 3**

**
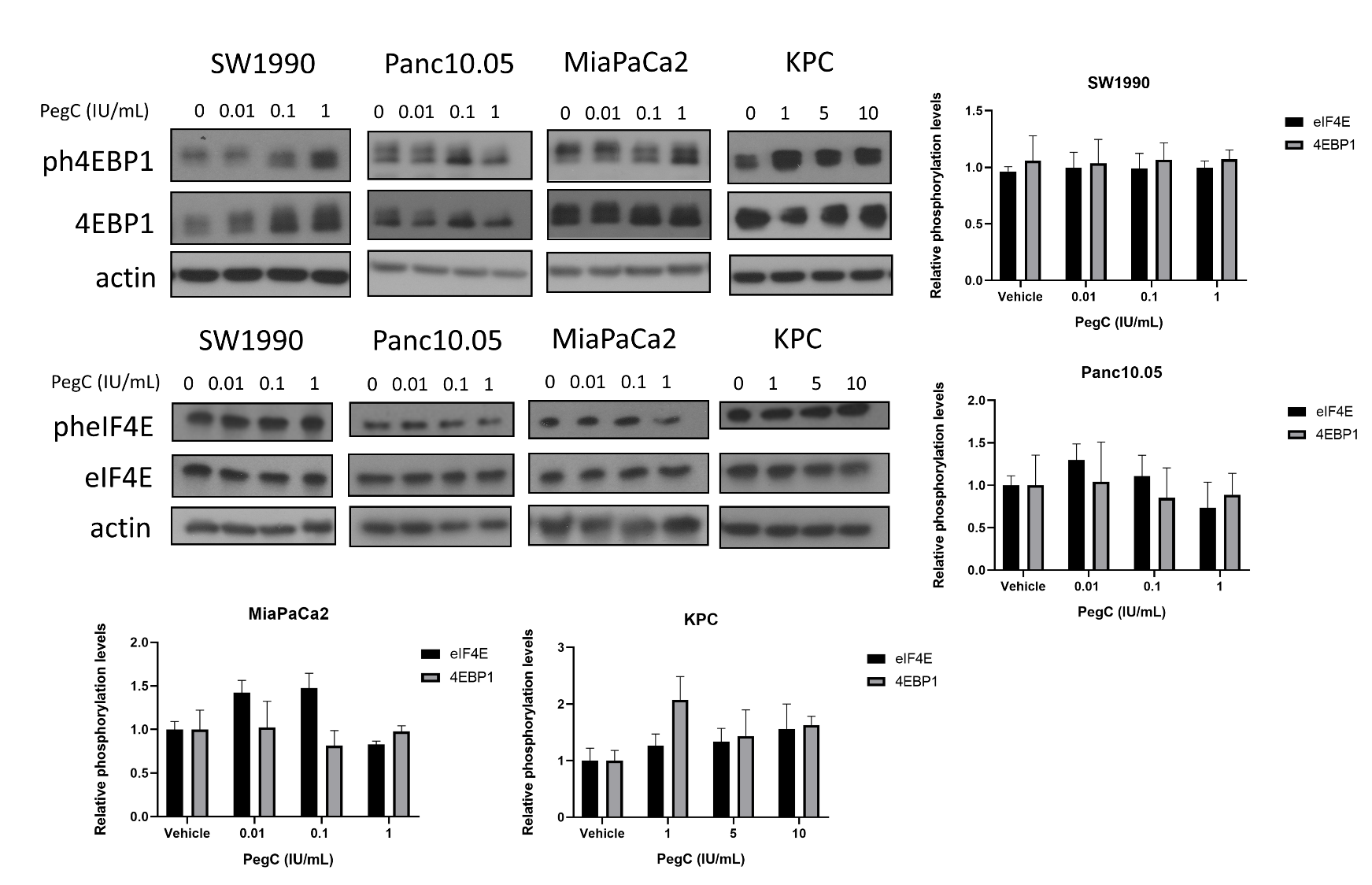
**

**Supplementary Figure 3: PegC does not impact mRNA translation machinery downstream of mTOR.** PDAC cell lines were treated with the indicated doses of PegC for 24h. Cell lysates were subjected to immunoblotting with the indicated antibodies and actin was used as a loading control. The results are expressed as relative phosphorylation levels and the bar diagrams represents densitometric quantification of three independent experiments normalized to the vehicle control.

**SUPPLEMENTARY FIGURE 4**

**
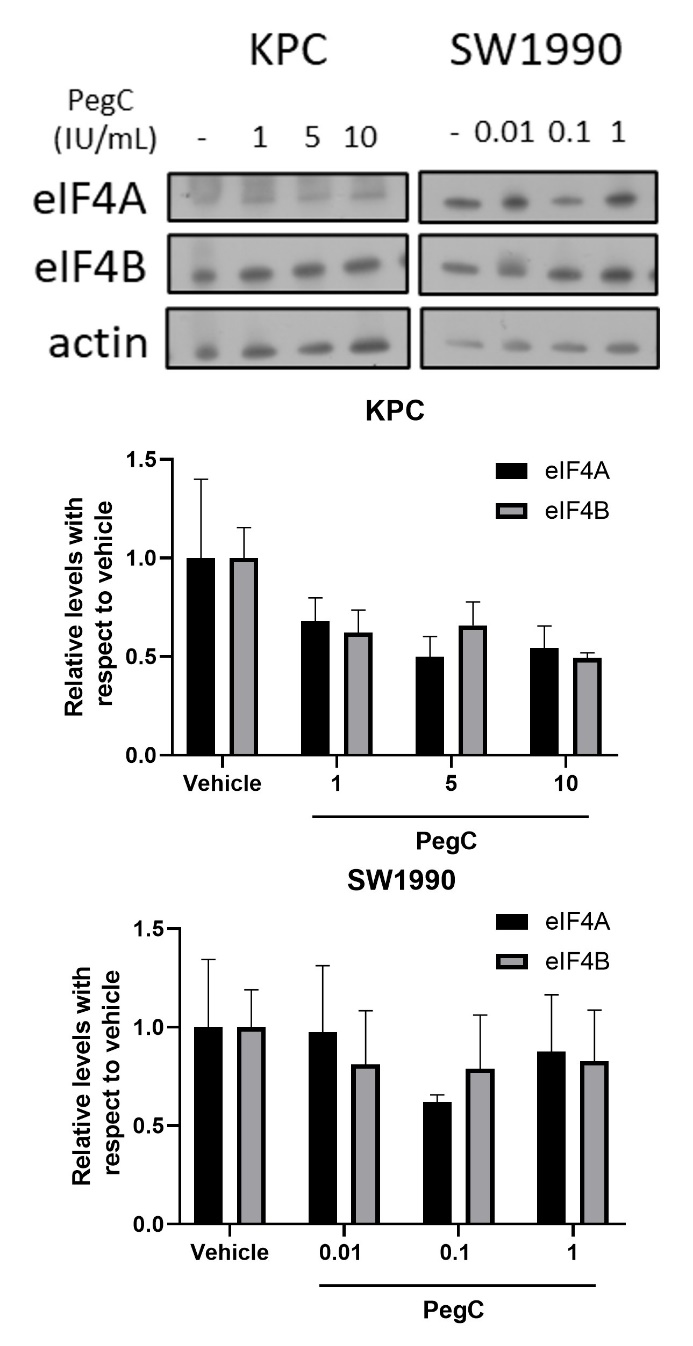
**

**Supplementary Figure 4: PegC does not inhibit cap dependent translation machinery.** KPC and SW1990 cells were treated with the indicated doses of PegC for 24h. After 24h, cell lysates were prepared and resolved by SDS-PAGE. Immunoblotting was performed for eIF4A and eIF4B with actin as a loading control. The bar diagrams represent densitometric quantification of three independent experiments normalized to the vehicle control.

**SUPPLEMENTARY FIGURE 5**

A

B

**Supplementary Figure 5: PegC does not potentiate the anti-cancer effect of cytotoxic or targeted drugs. (A)** KPC cells were treated with dose curves of the indicated chemotherapy alone or in combination with a low dose (IC_10-30_) of PegC. Cell proliferation was measured 72h after treatment by WST-1. **(B)** KPC and SW1990 cells were treated with dose curves of the indicated chemotherapy alone or in combination with a low dose (IC_10-30_) of PegC. Cell proliferation was measured 72h after treatment by WST-1. IC_50_ values were calculated using GraphPad Prism software. Results from at least three independent experiments are summarized in the tables and expressed as mean IC_50_ ± SD.
